# Identifying the Determinants of Anticipated Post-Pandemic Mode Choices in the Greater Toronto Area: A Stated Preference Study

**DOI:** 10.1177/03611981221145133

**Published:** 2023-01-12

**Authors:** Patrick Loa, Khandker Nurul Habib

**Affiliations:** 1 Department of Civil & Mineral Engineering, University of Toronto, Toronto, Canada

**Keywords:** mode choice, post-pandemic, COVID-19, stated preference, accessibility

## Abstract

The COVID-19 pandemic had a significant impact on travel mode choices in cities across the world. Driven by perceptions of risk and the fear of infection, the pandemic resulted in an increased preference for private vehicles and active modes and a reduced preference for public transit and ride-sourcing. As travel behavior and modal preferences evolve, a key question is whether the pandemic will result in long-term changes to travel mode choices. This study uses data from a web-based survey to examine the factors influencing mode choices for non-commuting trips in the post-pandemic era. Specifically, it uses stated preference data to develop a random parameter mixed logit model, which is used to compare the elasticity of key variables across different income and age groups. The results of the study highlight the influence of sociodemographic attributes and pre-pandemic travel habits on anticipated post-pandemic mode choices. Additionally, the results suggest that frequent users of private vehicles, public transit, and active modes are likely to continue to use these modes post-pandemic. Furthermore, the results highlight the potential for the perception of shared modes to influence post-pandemic mode choice decisions. The results of the study offer insights into policy measures that could be applied to address the increased use of private vehicles and reduced use of transit during the pandemic, while also emphasizing the need to ensure that certain segments of the population can maintain a sufficient level of mobility and access to transport.

The COVID-19 pandemic had a significant impact on activity and travel behavior, including participation in out-of-home activities and travel mode choice. As the world continues to recover from the effects of the pandemic and daily life progresses toward pre-pandemic normality, it remains to be seen whether the pandemic will have a long-term impact on travel behavior. Any such impact would have important implications for transportation planning in the post-pandemic era, particularly if the pandemic has changed travel habits and modality styles, which are aspects of an individual’s lifestyle that refer to the travel modes they habitually use (*1*). Previous studies have found that modality styles can influence short- and long-term decisions regarding activity participation and travel behavior, including residential location choice, mobility tool ownership, and mode choices (*2*). Consequently, it is essential to examine the potential determinants of post-pandemic mode choices and investigate whether the influence of said determinants differs across the population. Furthermore, exploring the influence of pre-pandemic travel habits on post-pandemic mode choices can offer insights into the potential similarities and differences between pre-pandemic and post-pandemic mode choices.

Travel mode choices have important implications for the transportation system and its users. Choices made by travelers play an important role in the short-term (e.g., service planning, road space allocation) and long-term (e.g., infrastructure investments, transportation master plans) management of the system. Additionally, the options (or lack thereof) available to individuals have an impact on their mobility and accessibility (*3*). The relative lack of accessibility can have important societal implications, because it is an “important prerequisite for social inclusion” (*4*) and has been found to influence well-being and quality of life (*5*). Traditionally, travel mode choices have been influenced by a combination of the attributes of the traveler, the characteristics of the trip, and the attributes of the transportation network (*6*). More recent studies have also highlighted the role of travel habits (*7*) and satisfaction (*8*) on mode choices. However, anticipated post-pandemic mode choices have (understandably) received relatively little attention thus far in the literature.

This study examines the factors influencing anticipated post-pandemic mode choices for non-commuting trips among residents of the Greater Toronto Area (GTA). The focus on non-commuting trips is motivated by the positive impact of maintenance and discretionary activities on both physical and emotional well-being (*9*, *10*) and the relative flexibility of the scheduling of these activities compared to mandatory activities (*6*). In the context of this study, the post-pandemic period refers to the period of time during which COVID-19 is no longer considered a public health threat. Using data from stated preference (SP) experiments regarding post-pandemic mode choices, this study develops a random parameter mixed logit model. Based on the final specification of the model, the direct and cross elasticities of key variables are calculated to explore their impact on mode choices in the post-pandemic period. The results of this study aim to provide insights into the factors influencing post-pandemic mode choices, including the influence of pre-pandemic travel behavior. Additionally, this information can help inform policies that aim to mitigate the shifts in modal preferences that resulted from the pandemic.

The remainder of the paper is organized as follows. First, a review of previous studies focusing on the impacts of COVID-19 on pandemic-era and anticipated post-pandemic travel mode choices are presented. Next, the design and administration of the survey are described, descriptive statistics of the survey sample are presented, and the process used to design the SP experiments is outlined. Then, the formulation and final specification of the empirical model are presented, and the results of the elasticity analysis are summarized. Finally, the implications of the results of the study are discussed.

## Literature Review

The disruptions caused by the COVID-19 pandemic have resulted in considerable effort being dedicated to studying its impact on travel behavior. Aside from participation in activities outside of the home, these studies often focus on the impact of the pandemic on modal preferences, utilizing either passive data or survey data. The studies have consistently found that the pandemic resulted in a reduced inclination to use shared modes, including public transit and ride-sourcing, and an increased inclination to use private vehicles and active modes (*11*, *12*). Examples of this shift are reported in Pase et al. (*13*) and Teixeira and Lopes (*14*), both of whom found evidence that bicycle sharing was being used as a substitute for public transit early in the pandemic. Similarly, Molloy (*15*) found that the pandemic resulted in a decline in the distance traveled by transit and an increase in the distance traveled using private vehicles and active modes. Survey-based approaches complement the findings of these studies by offering insights into the factors influencing the shift in modal preferences resulting from the pandemic.

The modal shifts that have resulted from the pandemic have been attributed to attitudes toward the pandemic and perceptions of risk. Specifically, studies on the topic have typically found that attitudes toward public transit and ride-sourcing have become more negative. In contrast, attitudes toward private vehicles and active modes have either been unaffected or have become more favorable (*16*, *17*). In the literature, the changes in attitudes and perceptions resulting from the pandemic are often ascribed to the perceived risk of infection and the fear of infection associated with the use of each mode. In particular, previous studies have found that the decision to use public transit during the pandemic was affected by the perceived risk of infection (*18*) and the perception of safety (*19*). It is clear that the onset of the pandemic resulted in a shift in modal preferences; however, it remains to be seen whether these shifts will remain in the post-pandemic period. Studies examining the evolution of modal preferences during the pandemic typically find that the utilization of private vehicles and active modes has rebounded more strongly than public transit (*15*, *20*). Notably, there appears to be evidence that the recovery of ride-sourcing usage is outpacing that of transit usage, which can partly be attributed to the reluctance of certain individuals to use public transit (*21*, *22*).

In addition to descriptive analysis, mode choice decisions during the pandemic have also been examined through the use of econometric models. Aside from the typical determinants of mode choices (e.g., travel time and travel cost), studies on the topic often find that mode choices during the pandemic were influenced by pre-pandemic travel behavior. For example, Costa et al. (*23*) reported that those who used ride-sourcing on a frequent basis in Brazil pre-pandemic were more likely to identify these services as their primary travel mode during the pandemic. A similar relationship between pre-pandemic travel behavior and the likelihood of choosing a mode for shopping trips in India and Bangladesh is reported by Zannat et al. (*24*). Moreover, Awad-Núñez et al. (*25*) found that the willingness to use shared modes (e.g., transit, ride-sourcing, taxi) following the initial lockdowns in Spain was affected by sociodemographics, mobility habits, and the implementation of health and safety measures. Additionally, it is evident that mode choices during the pandemic were influenced by perceptions of risk. For example, using a hybrid choice model, Aaditya and Rahul (*26*) found that the awareness of COVID-19 was positively associated with the propensity to travel in a private vehicle, and that perceived safety increased the likelihood of an individual using transit in India. Using a similar approach, Scorrano and Danielis (*27*) found that individuals who were less risk averse were more likely to continue using shared modes during the early stages of the pandemic in Trieste, Italy. Moreover, using SP data, Zhang et al. (*28*) reported that the perceived severity of COVID-19 was negatively associated with the propensity to use transit and shared ride-sourcing in Beijing.

Aside from travel behavior during the pandemic, a limited number of studies have investigated the potential nature of post-pandemic modal preferences. Although these studies offer insights into post-pandemic travel behavior, they tend to focus on whether a specific mode will be used. For example, using protection motivation theory, Mashrur et al. (*29*) found that the tendency to adopt protective measures against COVID-19 reduced the likelihood that an individual would use public transit post-pandemic. Similarly, Javadinasr et al. (*30*) found that individuals in the U.S.A. who believed there was a high risk of contracting COVID-19 while using transit displayed a greater propensity to use transit less often post-pandemic than they did pre-pandemic. Moreover, descriptive studies tend to find that certain pre-pandemic transit and ride-sourcing users will not return to these services post-pandemic (*31 [Bibr bibr32-03611981221145133] [Bibr bibr33-03611981221145133]*–*34*).

Despite these efforts, additional work is required to understand the determinants of post-pandemic mode choices better. To date, studies on the topic have focused more on understanding whether a mode will be used, rather than examining the factors influencing the decision to use a certain mode for a given trip, as outlined in Table 1. To the authors’ knowledge, there has as yet been no study that examines the determinants of travel mode choices through the lens of random utility theory. Consequently, relatively little is known about the extent to which personal attributes, trip-related attributes, and perceptions of risk influence the decision to use a given mode for a specific trip. This is a significant research gap, because understanding the determinants of post-pandemic mode choices will be essential for transportation planning and policy decisions. This study aims to contribute to the literature by analyzing the determinants of post-pandemic mode choices through the estimation of an econometric model. Specifically, it uses SP data collected through a web-based survey to estimate a random parameter mixed logit model of travel mode choices for non-commuting trips. In addition to offering insights into post-pandemic model preferences, the results of the study can also shed light on the extent to which mode choices are affected by perceptions of risk and pre-pandemic travel behavior.

**Table 1.** Outcomes of Interest of Post-Pandemic Mode Choice Studies

Study area	Study period	Definition of “post-pandemic”	Outcomes of interest (method)	Reference
Beijing	November 27 to 30, 2020	Resumption of in-person education and economic activities	Decision to use public transit (structural equation model)	Zhao and Gao (*18*)
Spain	April 28 to May 12, 2020	After the initial lockdowns in Spain	Willingness to use and pay for shared modes (two-step Heckman model)	Awad-Núñez et al. (*25*)
Gdańsk	May and June, 2020	After the pandemic situation has stabilized	Willingness to return to public transit (descriptive analysis)	Przybylowski et al. (*32*)
Greater Toronto Area	July 10 to 28, 2022	COVID-19 is no longer considered a threat	Decision to abstain from using transit and decision to maintain or increase transit frequency (structural equation model)	Mashrur et al. (*29*)
U.S.A.	April to October, 2020 and November 2020 to May 2021	COVID-19 is no longer considered a threat	Post-pandemic transit frequency (random effects ordered probit model)	Javadinasr et al. (*30*)
Greater Melbourne	June 26 to August 8, 2020	COVID-19 is no longer considered a threat	Volume and share of commute modes (descriptive analysis)	Currie et al. (*31*)
Greater Toronto Area	July 2020	COVID-19 is no longer considered a threat	Decision to continue using ride-sourcing post-pandemic (descriptive analysis)	Loa et al. (*33*)
U.S.A.	July and August, 2020	COVID-19 is no longer considered a threat	Decision to use various shared modes post-pandemic (descriptive analysis)	Menon et al. (*34*)

## Data and Methods

### Survey Design and Administration

The data used in this study were collected as part of the Study into the Use of Shared Travel Modes (SiSTM), whose primary goal was to understand the impact of the COVID-19 pandemic on ride-sourcing in the GTA. As part of the SiSTM, two cycles of a web-based survey were conducted, one in July 2020 and another in July 2021. In the SiSTM surveys, respondents were asked (among other things) to answer a series of questions with regard to their sociodemographic attributes, travel behavior before and during the pandemic, and their attitudes toward the pandemic. Additionally, they were asked to complete a series of SP experiments pertaining to mode choice for commuting and non-commuting trips during and after the pandemic. In the survey, the post-pandemic period was defined as the period during which COVID-19 is no longer a public health threat.

**Figure 1.**
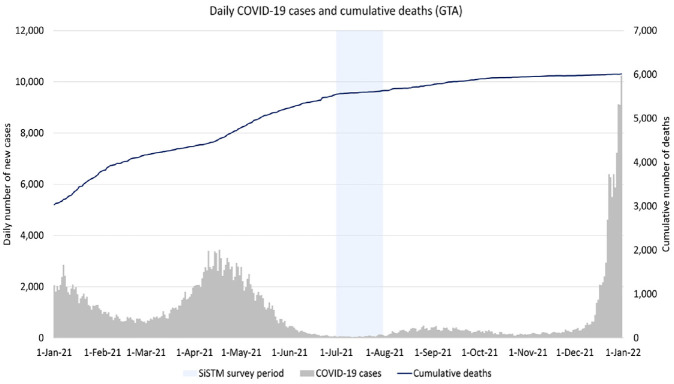
COVID-19 case counts and cumulative deaths in the GTA. *Note*: GTA = Greater Toronto Area; SiSTM = Study into the Use of Shared Travel Modes.

**Figure 2.**
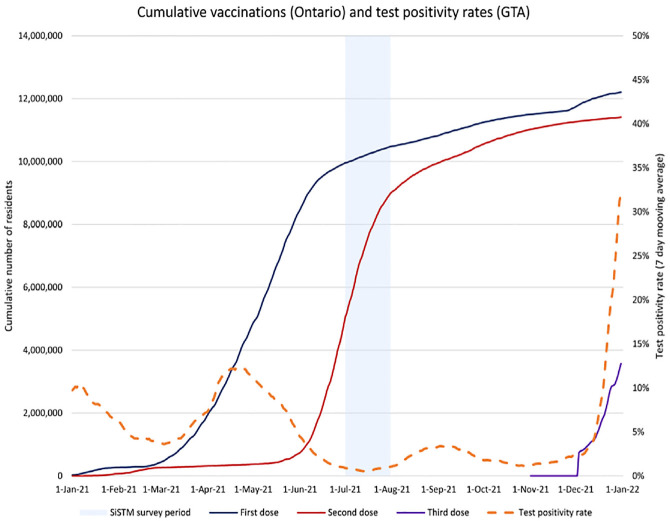
Vaccine doses administered in Ontario and COVID-19 test positivity rates in the GTA. *Note*: GTA = Greater Toronto Area; SiSTM = Study into the Use of Shared Travel Modes.

This study uses data collected through the second cycle of the SiSTM survey. As shown in Figure 1, the second cycle was conducted during a period in which the number of new COVID-19 cases being reported was relatively low. As a result, the public health measures that were in place during the survey period were relatively relaxed compared with periods that had higher case counts. The second cycle was also conducted at a time during which the number of Ontario residents receiving their second dose of the COVID-19 vaccines available in the province was on the rise and test positivity rates were relatively low (as shown in Figure 2).

The SiSTM survey was coded into a web-based survey interface, and a link to access the survey was sent to a market research company. The company then invited a random sample of the members of its consumer panel to participate in the survey, providing non-monetary compensation upon completion of the survey. As part of the administration of the survey, a residential location quota was imposed to help ensure that the distribution of the home locations of the survey respondents was consistent with that of the study area. The minimum required sample size for the survey was informed by the standard error of the experimental design, the sample size standards for SP studies outlined in Rose and Bliemer (*35*), and the sample sizes of similar studies in the literature. After the data were cleaned, the final sample size was 806 completed responses; see Loa et al. (*36*) for more information. This sample size is consistent with the recommended minimum sample size values outlined by Orme (*37*) (83 for this study) and Lancsar and Louviere (*38*) (20 responses per SP scenario).

### Descriptive Statistics

The distributions of key personal and household attributes are summarized and compared with the 2016 Canadian census in Table 2. Compared with the study area, women are slightly overrepresented in the sample, as are the residents of Toronto, Peel Region, and York Region. Given that public transit usage is much more prevalent in Toronto compared with the rest of the study area (*39*), this could result in members of the sample being more inclined toward the use of transit compared with the population as a whole. Individuals between the ages of 20 and 45 are also overrepresented in the sample, which likely stems from the use of a web-based survey to collect data and the administration of the survey to a market research panel. Similarly, individuals from households earning between $50,000 and $100,000 annually are overrepresented in the sample, whereas individuals from other income levels are underrepresented.

**Table 2.** Comparison of Key Sociodemographic Attributes—SiSTM Survey versus Census

	SiSTM survey	2016 census
Gender (%)		
Male	40.2	48.5
Female	58.8	51.5
Another gender identity	1.0	0.0
Age (%)		
19 and under	3.0	22.9
20–24	9.7	6.9
25–29	12.2	7.0
30–34	11.9	7.0
35–39	10.8	6.8
40–44	8.7	7.0
45–49	7.7	7.4
50–54	8.8	7.8
55–59	7.4	6.9
60–64	6.7	5.6
65 and over	13.2	14.7
Household income (%)		
Under $50,000	27.4	30.7
$50,000–$100,000	36.6	30.9
$100,000 and above	27.3	38.4
Prefer not to answer	8.7	0.0
Home location (%)		
Toronto	45.2	42.6
York	17.9	17.3
Durham	4.8	10.1
Peel	23.0	21.5
Halton	9.2	8.5
Mobility tool ownership		
Possesses a driver’s license	85.9%	na
Has access to private vehicle	87.1%	na
Possesses a transit pass	40.8%	na
Possesses at least one adult bicycle	62.4%	na

*Note*: SiSTM = Study into the Use of Shared Travel Modes; na = not applicable.

**Figure 3.**
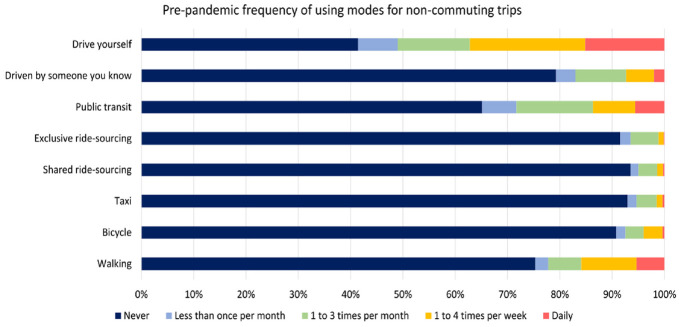
Summary of modes used for non-commuting trips before the pandemic.

**Figure 4.**
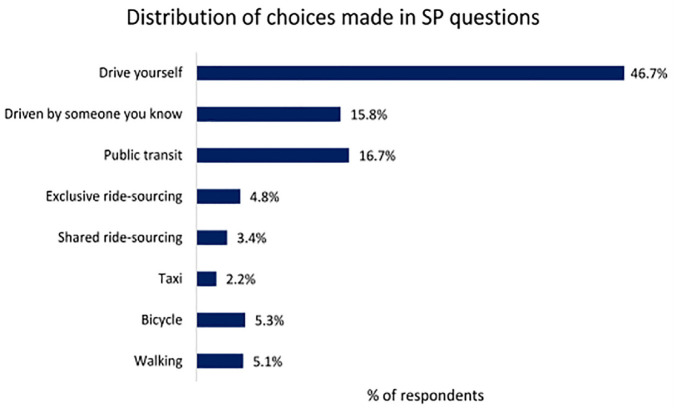
Distribution of responses to post-pandemic SP questions for non-commuting trips. *Note*: SP = stated preference.

As part of the SiSTM survey, respondents were asked to indicate the frequency with which they used various modes of travel before the pandemic for commuting and non-commuting trips. As shown in Figure 3, driving was the mode used most frequently for non-commuting trips, followed by public transit and walking. Conversely, taxi, ride-sourcing, and bicycle were the modes that were least likely to be used by the respondents for non-commuting trips. This is relatively consistent with the choices made in the SP experiments, as shown in Figure 4. Interestingly, the percentage of respondents choosing to use public transit and to be driven by someone they know is roughly equal. In addition, the percentage of people choosing transit in the SP experiments is lower than that of the percentage of respondents indicating that they used transit in the pre-pandemic period.

**Figure 5.**
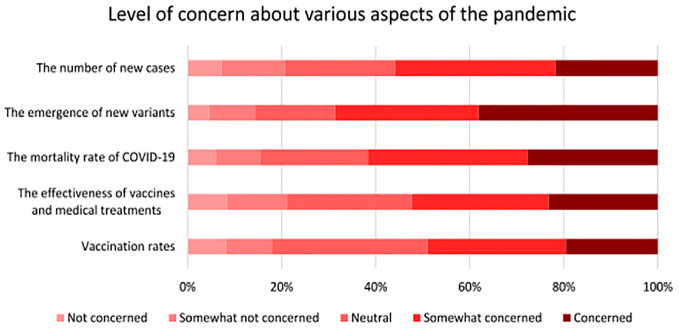
Concerns about different aspects of the pandemic among respondents.

**Figure 6.**
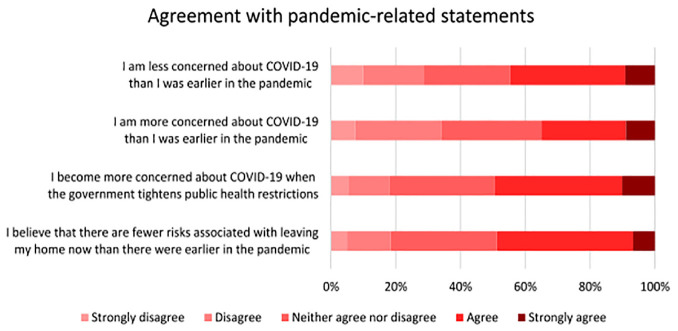
Level of agreement with statements regarding changes in concern and perceptions of risk.

At the time that the second cycle of the SiSTM survey was conducted, the Alpha, Beta, Gamma, and Delta variants of the SARS-CoV-2 virus had been identified (*40*). To help account for this, respondents who took part in the second cycle survey were asked a series of attitudinal questions pertaining to their level of concern about different aspects of the pandemic (including the emergence of new variants). As shown in Figure 5, over two-thirds of respondents expressed concerns about the emergence of new variants, whereas roughly 62% expressed concerns about the mortality rate of COVID-19. Additionally, respondents were asked to indicate their level of agreement with statements comparing their level of concern toward COVID-19 at the time of the survey with their concern earlier in the pandemic. As outlined in Figure 6, roughly half of the respondents indicated that they were less concerned about COVID-19 than they were earlier in the pandemic, and that there were fewer risks associated with leaving their home than earlier in the pandemic.

### Experimental Design

The SP experiments were designed to gain insights into the determinants of post-pandemic mode choice decisions. The alternatives and their corresponding attribute values are summarized in Table 3. The alternatives included in the SP experiments were chosen based on the travel modes available in the study area. In contrast, the attributes were chosen according to a review of similar studies. The *drive yourself* and *driven by someone you know* modes were only available to respondents who indicated that they had access to a private vehicle as a driver or passenger. The SP experiments were based on a hypothetical non-commuting trip, and the trip length corresponding to each mode was determined based on the most recent iteration of the regional household travel survey, the Transportation Tomorrow Survey (TTS) (*41*). Four sets of modes were considered: motorized modes (i.e., private vehicle, taxi, ride-sourcing), public transit, bicycle, and walking. The trip distances for the motorized and public transit modes were defined based on the average values from the TTS, whereas those for the bicycle and walking modes were defined as the 95th percentile values. The discrepancy in the methods used to determine the trip distance corresponding to each mode stemmed from the desire to ensure that the travel times of the alternatives were relatively competitive with one another.

**Table 3.** Attributes of the Alternatives Included in the SP Experiments

Attribute	Alternative	Value(s)
Travel time (min)	Drive yourself	10.9, 12.1, 15.8, 21.4
	Driven by someone you know	10.9, 12.1, 15.8, 21.4
	Public transit	22.8, 32.6, 35.8, 40.7
	Exclusive ride-sourcing	10.9, 12.1, 15.8, 21.4
	Shared ride-sourcing	10.9, 12.1, 15.8, 21.4
	Taxi	10.9, 12.1, 15.8, 21.4
	Bicycle	19.6, 26.2, 28.8, 32.7
	Walking	30.7, 38.4, 42.2, 46.1
Travel cost ($)	Drive yourself	2.05, 4.10, 6.16, 7.18
	Driven by someone you know	1.64, 2.05, 2.46, 3.08
	Public transit	3.25
	Exclusive ride-sourcing	11.17, 13.96, 16.76, 22.34
	Shared ride-sourcing	8.94, 11.17, 13.41, 17.87
	Taxi	18.13, 22.66, 27.20, 34.00
Parking cost ($)	Drive yourself	5, 10, 15
Waiting time (min)	Public transit	2, 5, 8, 10
	Exclusive ride-sourcing	2, 5, 8, 12
	Shared ride-sourcing	2, 5, 8, 12
	Taxi	2, 5, 8, 12
Walking time (min)	Public transit	1, 5, 12
Number of other passengers	Shared ride-sourcing	0, 1, 2
Level of crowding	Public transit	No crowding, moderately crowded, very crowded
All passengers are required to wear masks	Public transit	Yes, No
	Exclusive ride-sourcing	Yes, No
	Shared ride-sourcing	Yes, No
	Taxi	Yes, No
Vehicles are disinfected at the end of each day	Public transit	Yes, No
	Exclusive ride-sourcing	Yes, No
	Shared ride-sourcing	Yes, No
	Taxi	Yes, No
There is a physical barrier between the driver and passengers	Public transit	Yes, No
	Exclusive ride-sourcing	Yes, No
	Shared ride-sourcing	Yes, No
	Taxi	Yes, No

*Note*: SP = stated preference.

Based on these reference values, the baseline values of the travel time and cost attributes were calculated. The baseline values were then modified to create a set of levels for each attribute. The travel time for motorized modes was calculated based on an assumed speed of 45 km/h, whereas that for public transit was based on an assumed speed of 40 km/h; both speed values were based on the experimental design outlined in Weiss et al. (*42*). Similarly, the travel time for bicycles and walking was calculated based on assumed speeds of 15 km/h and 4 km/h, respectively (*43*, *44*). The waiting time for the *public transit* mode was determined based on the headways of the transit services offered in the study area. In contrast, the waiting times for the *taxi* and *ride-sourcing* modes were selected to mirror that of public transit because of the lack of available information. The walking time associated with the *public transit* mode was defined according to a combination of the service standards in the study area and the standard values for the maximum walking distance to access transit (400 m and 800 m) (*45*).

Similarly, the cost associated with each mode was determined according to an assumed per-kilometer and/or per-minute cost. The cost of the *drive yourself* mode was based on the annual per-kilometer cost of owning a compact automobile published by the Canadian Automobile Association. Based on the assumption that the vehicle is driven 20,000 km annually, the cost was listed as $ 0.37/km (*46*); for the *driven by someone you know* mode, the cost was defined as 50% of this value, based on the work of Bhat and Sardesai (*47*). The cost of the *public transit* mode was defined as the fare for adults using the services offered by the Toronto Transit Commission, given its relatively high ridership compared with the other services in the area. For *exclusive ride-sourcing*, the travel cost was determined according to the $ 0.81/km and $ 0.18/min rate cited in Weiss et al. (*42*). The cost of *shared ride-sourcing* was taken as 80% of the cost of exclusive ride-sourcing, based on Brown (*48*). Finally, the cost of using a taxi was calculated according to the rates posted by one of the taxi companies that operate in the study area: $3.25 plus $1.75/km and $ 0.5 per minute spent waiting for the traveler (*49*). Because of the lack of publicly available information on parking costs in the study area, round numbers were used for the parking cost attribute.

In addition to the attributes in Table 3, the experimental design also included two contextual variables: the number of doses of a COVID-19 vaccine received by the respondent; and whether mass vaccination had been achieved. The D-efficient approach was used to design the SP experiments, and Ngene software was used to produce the combinations of attributes that would be shown to respondents (i.e., choice situations). D-efficient design aims to produce experimental designs that, when used to estimate a statistical model, yield parameters with standard errors that are as low as possible (*50*). However, efficient design methods require the researcher to provide a set of parameter values corresponding to each attribute that will be included in the design (i.e., the priors) and specify a model formulation. For this survey, prior values with the correct sign were obtained from a previous SP survey outlined in Loa et al. (*51*) that was conducted in the city of Toronto, and a multinomial logit model (MNL) was used. For attributes that were not included in this study, the parameters were assumed to have relatively low magnitudes, and previous studies were used to determine the appropriate signs. The design process produced 12 choice situations for each of the two possible choice sets, and each respondent was shown three at random. The econometric model that was estimated as part of this study was developed using 2,418 choice observations.

## Empirical Model and Results

### Model Formulation

The respondents’ post-pandemic mode choices were modeled using a mixed logit model. Compared with the MNL model, mixed logit models can accommodate preference heterogeneity, correlations among unobserved factors, and unrestricted substitution patterns between alternatives (*52*). This study uses the data collected from the SiSTM survey to develop a random parameter mixed logit model, where parameters can be defined as a distribution rather than a deterministic value, allowing the model to capture variations in tastes (*52*). Let $U_{i,j}$ represent the utility obtained by person i when they choose alternative j . This utility can be decomposed to a systematic component that can be modeled by the researcher $\left(V_{i,j}\right)$ and a random component that cannot be observed $\left(\varepsilon_{i,j}\right)$ . The systematic component of utility can be modeled as a function of the attributes of the alternative and the characteristics of the decision-maker:

(1) $$V_{i,j}=\beta^{\prime }x+\Gamma^{\prime }z\beta_{0,j}$$

where

β is a vector of deterministic parameters

$\beta_{0,j}$ is the constant associated with alternative j

x,z are vectors of explanatory variables

Γ is a vector of random parameters

The random parameter mixed logit model developed in this study uses the same choice probability as the MNL model. However, because the model includes random parameters, the probability of person i choosing alternative j is defined as (*52*):

(2) $$P_{i}\left(j\right)=\int \frac{\exp\left(\mu V_{i,j}\right)}{\sum_{k\in C_{i}}\exp\left(\mu V_{i,k}\right)}f\left(\Gamma \right)d\Gamma$$

where

μ is the scale parameter

$C_{i}$ is the set of alternatives available to person i

k is the index of available alternatives

f(Γ) is the probability of the density of the random parameters

As part of the SiSTM survey, each respondent was asked to complete three choice experiments. Consequently, let $P_{i}$ represent the probability of person i choosing alternatives $j_{1}$ , $j_{2}$ , and $j_{3}$ in choice situations 1, 2, and 3, respectively:

(3) $$P_{i}=P_{i}\left(j_{1}\right)*P\left(j_{2}\right)*P\left(j_{3}\right)=\overset{3}{\underset{t=1}{\Pi }}P_{i}\left(j=j_{t}\right)$$

where

t is the index of choice situations

The likelihood function of the model is given by:

(4) $$L\left(\beta ,\Gamma ,x,z\right)=\overset{N}{\underset{i=1}{\Pi }}P_{i}=\;\overset{N}{\underset{i=1}{\Pi }}\overset{3}{\underset{t=1}{\Pi }}P_{i}\left(j=j_{t}\right)$$

where

N is the number of survey respondents (806)

The random parameter mixed logit model was estimated using the Apollo package written for the statistical computing software R (*53*). The package accounts for correlations among choices made by the same individual and calculates the robust standard error of the parameters accordingly.

### Final Specifications

The final specification of the random parameter mixed logit model is summarized in Table 4 under the heading “Final model.” During the model estimation process, variables corresponding to sociodemographic attributes, attitudinal information, and SP attributes were tested. Additionally, the frequency with which an individual used various modes of travel before the pandemic was included in the model. Specifically, the response options outlined in Figure 1 were coded numerically from 0 (never) to 4 (daily). Taking such an approach to include this information in the model implies that the effects of these variables are linear. The decision to retain a variable in the model was made based on the sign and robust *t*-statistic of the corresponding parameter; however, a small number of variables that were not statistically significant were retained because of the insights they offered. The adjusted rho-squared value of the model was 0.507, which is indicative of a good model fit. As part of the model estimation process, various nesting structures were tested. However, each of the nesting structures produced a logsum parameter that was either greater than 1 or not statistically different from 1 at the 95% confidence level. Consequently, the MNL choice probability was used in the model in the interests of parsimony.

**Table 4.** Final Model Specifications

Variable	Final model		Health risk model	
	Estimate	Robust *t*-statistic	Estimate	Robust *t*-statistic
Drive yourself				
Travel time (min)—mean	−1.712	−7.018	−1.713	−7.007
Travel time (min)—SD	1.453	6.023	1.455	6.045
Parking cost ($)	−0.256	−5.130	−0.253	−5.081
Age (years)	0.041	2.805	0.041	2.813
Person possesses a driver’s license (1 if yes, 0 otherwise)	1.540	1.672	1.536	1.652
Frequency of driving before the pandemic	1.414	7.626	1.411	7.593
Driven by someone you know				
Constant	−1.273	−0.824	−1.217	−0.780
Travel time (min)—mean	−1.131	−2.949	−1.131	−2.893
Travel time (min)—SD	2.321	4.536	2.294	4.529
Frequency of being driven before the pandemic	1.152	4.412	1.148	4.292
Public transit				
Constant	−2.720	−2.000	−2.665	−1.944
Out-of-vehicle travel time (min)	−0.027	−2.009	−0.024	−1.744
Person belongs to a household that earns less than $50,000 annually (1 if yes, 0 otherwise)	0.459	1.792	0.474	1.852
Person indicated they will be less willing to use shared modes post-pandemic than they were pre-pandemic	−0.740	−3.003	−0.725	−2.939
Frequency of using public transit before the pandemic	0.263	3.127	0.263	3.112
Level of crowding: very crowded (1 if yes, 0 otherwise)	na	na	−0.205	−1.233
Exclusive ride-sourcing				
Constant	−3.099	−2.231	−3.223	−2.296
Age (years)	−0.031	−2.879	−0.031	−2.872
Person possesses a driver’s license (1 if yes, 0 otherwise)	1.049	2.691	1.075	2.769
Frequency of exclusive ride-sourcing usage before the pandemic	0.708	4.282	0.712	4.258
Vehicles are disinfected at the end of each day (1 if yes, 0 otherwise)	na	na	0.240	1.078
Shared ride-sourcing				
Constant	−0.060	−0.037	0.287	0.175
Travel time (min)—mean	−2.030	−2.402	−1.905	−3.009
Travel time (min)—SD	1.735	1.796	1.451	2.140
Age (years)	−0.048	−3.622	−0.048	−3.621
Person indicated they are concerned about the daily number of new COVID-19 cases in Ontario (1 if yes, 0 otherwise)	−1.083	−2.598	−1.100	−2.673
Number of other passengers	na	na	−0.132	−0.735
Taxi				
Constant	−2.086	−1.483	−2.291	−1.584
Age (years)	−0.042	−2.319	−0.041	−2.333
Physical barrier between driver and passengers (1 if yes, 0 otherwise)	na	na	0.425	1.316
Bicycle				
Constant	−5.779	−4.022	−5.745	−3.969
Person belongs to a household that earns more than $100,000 annually (1 if yes, 0 otherwise)	−1.378	−2.393	−1.378	−2.416
Number of household adult bicycles	0.194	1.258	0.196	1.289
Frequency of cycling before the pandemic	1.033	4.829	1.030	4.827
Walking				
Constant	−0.162	−0.103	−0.248	−0.157
Travel time (min)—mean	−1.310	−7.667	−1.321	−7.570
Travel time (min)—SD	0.659	5.657	0.671	5.677
Frequency of walking before the pandemic	1.379	5.421	1.379	5.418
Generic parameters				
Travel cost ($)—mean	−2.576	−7.359	−2.582	−7.326
Travel cost ($)—SD	1.868	11.399	1.872	11.344
Goodness-of-fit measures				
Number of observations	2,418		2,418	
Final loglikelihood	−2405.3		−2401.5	
$\rho^{2}\;\mathrm{value}\;\mathrm{against}\;\mathrm{null}\;\mathrm{model}\;\left(\mathrm{adjusted}\;\rho^{2}\right)$	0.514 (0.507)		0.515 (0.507)	
$\rho^{2}\;\mathrm{value}\;\mathrm{against}\;\mathrm{constant}-\mathrm{only}\;\mathrm{model}$	0.327		0.328	

*Note*: SD = standard deviation; na = not applicable. The mean and standard deviation of each random parameter is reported separately; the random parameters were defined to follow the log-normal distribution. Travel cost was included in all utility functions, except for the *driven by someone you know*, *bicycle*, and *walking* alternatives.

Notably, the inclusion of the SP attributes corresponding to health and safety was not supported by the sign and *t*-statistics of the parameters. This could stem from the post-pandemic period being characterized as the period of time during which COVID-19 is no longer considered a public health threat, which may have lessened the impact of these attributes on mode choice decisions. Additionally, the absence of these attributes from the final model may be the result of changes in the perceived risk of COVID-19 and changes in attitudes towards the use of certain modes. However, these findings could also suggest that the inclusion of health and safety measures post-pandemic will not be sufficient to encourage the use of shared modes. To help explore the potential impact of these SP attributes on post-pandemic mode choices, a modified version of the final model was estimated; the final specification is presented under the “Health risk model” heading in Table 4.

The random parameters included in the model were specified as log-normal variables, because they were used to capture the influence of travel time and cost on travel mode choices. As Train (*52*) outlined, the log-normal distribution is useful in situations in which there is a prior expectation about the sign of the parameter. Consequently, each of the travel time and cost parameters were defined according to the log-normal distribution, given that the established standard is for these parameters to be negative. The results highlight the influence of travel costs on post-pandemic mode choices, particularly with regard to the decision to drive. In addition, the results also highlight the variation in the sensitivity to travel costs among members of the sample. Similar variations are also observed for travel time with regard to the *drive yourself*, *driven by someone you know*, *shared ride-sourcing*, and *walking* modes. Notably, the decision to use public transit was affected by out-of-vehicle travel time (defined as the sum of walking and waiting time) but was not affected by in-vehicle travel time. This could stem from the tendency for transit users to place a greater weight on walking and waiting time than in-vehicle travel time (*54*).

Aside from the attributes of the alternatives, sociodemographic attributes were also found to influence post-pandemic mode choices. In particular, the age of the respondents was found to influence the decision to drive oneself or use hailed modes of travel (i.e., taxis and ride-sourcing). This is consistent with previous studies, which have found that older individuals tend to favor travel in private vehicles. However, these results also contradict the work of Ozonder and Miller (*55*), who found that older individuals in the study area preferred taxis to ride-sourcing pre-pandemic. This shift may suggest that older individuals have become more reluctant to use shared mobility services because of the pandemic. In comparison, ride-sourcing use tends to be more prevalent among younger individuals (*56*, *57*). In addition, possessing a driver’s license increases the likelihood of an individual choosing the *drive yourself* and *exclusive ride-sourcing* alternatives. This could reflect the inclination of private vehicle owners toward travel reported by Rayle et al. (*58*) in their study on ride-sourcing use in San Francisco. Household income was also found to influence the propensity to choose public transit. This is expected, given that lower-income individuals tend to be more likely to rely on public transit (*59*).

Despite the absence of pandemic-related SP attributes in the final model, the results suggest that perceptions of risk also have the potential to affect post-pandemic mode choices. Specifically, those who indicated they would be less willing to use shared modes compared with before the pandemic were less likely to choose public transit. This reluctance, which was also reported in Currie et al. (*31*) and Anke et al. (*60*), could impair the recovery of transit ridership to pre-pandemic levels and pose challenges to transit agencies in the future. In contrast, those who indicated they were concerned about the daily reported number of new COVID-19 cases were less likely to choose shared ride-sourcing. Given the potential benefits of shared ride-sourcing, a decline in the use of this service could have a detrimental impact on congestion and emissions, particularly if shared ride-sourcing trips are replaced with trips made by exclusive ride-sourcing. These findings, coupled with the influence of perceived risks on mode choices during the pandemic, suggest that certain individuals may reduce their use of shared modes in the post-pandemic era.

Furthermore, pre-pandemic travel behavior was found to affect post-pandemic mode choices. Specifically, using a given mode frequently before the pandemic increased the likelihood that the mode was chosen in the SP experiments. This finding suggests that the extent to which a person used a given mode before the pandemic offers insights into whether they will continue to use the mode in the post-pandemic period. Previous studies have found that even when faced with changes in the decision context, habits formed before the change can still influence behavior following it (*18*). Moreover, similar evidence of the inertia effect has been reported in studies using revealed preference data, including Zannat et al. (*24*) and Bhaduri et al. (*61*). However, it is important to note that using SP data means that the decisions made in the choice situations may not fully reflect people’s observed behaviors (*62*). In addition, even if an individual continues to use a mode in the post-pandemic period, they could change the frequency with which they use this mode. Such a change could have important planning implications, particularly if it increases the use of private vehicles. Despite these results, there is also evidence that a subset of pre-pandemic transit users (*32*) and ride-sourcing users (*33*) will refrain from using these services in the post-pandemic era.

Based on the final specification of the *health risk model*, it appears that the implementation of public health measures may influence the decision to use exclusive ride-sourcing and taxis. In particular, the disinfection of vehicles at the end of each day increased the likelihood of exclusive ride-sourcing being chosen, whereas the presence of a barrier between the driver and passengers had a similar impact on taxi being chosen. The discrepancy in public health measures between the two modes could stem from the use of private vehicles to deliver ride-sourcing services, or differences in attitudes toward taxi and ride-sourcing services. Additionally, the results of the *health risk model* indicate that public transit was less likely to be chosen when vehicles were very crowded. Although crowding was found to deter the use of public transit pre-pandemic, there is evidence that its impact increased as a result of the pandemic (*63*, *64*). Finally, the number of other passengers in the vehicle was negatively associated with the likelihood of choosing shared ride-sourcing. Given the reduced willingness to share ride-sourcing trips with strangers that resulted from the pandemic (*65*), this may be indicative of less willingness to use shared ride-sourcing in the post-pandemic period, despite the reduced fares.

## Elasticity Analysis

Although the model results help provide insights into the determinants of post-pandemic mode choices, they primarily indicate whether a variable increases or decreases the probability of an alternative being chosen. Direct and cross elasticities were calculated to help understand and quantify the extent to which changes in key variables affect mode choices. The elasticities corresponding to two variables were calculated: parking cost of the *drive yourself* mode (because of an increase in driving during the pandemic) and out-of-vehicle travel time for the *public transit* mode (because of the impact of the pandemic on ridership). The analysis is comprised of two components: an analysis of the elasticities of the sample as a whole; and a comparison of the elasticities of different population segments (based on age and household income). Despite the discrepancies in the distribution of sociodemographic attributes between the sample and the census (as outlined in Table 1), statistical adjustments were not applied before the conduct of the elasticity analysis.

### Sample-Level Analysis

The direct and cross elasticities for the sample were aggregated using sample enumeration and are summarized in Table 5. The results suggest that increasing parking costs would have the greatest impact on the use of ride-sourcing, taxis, and public transit. The shift from private vehicles to taxis and ride-sourcing vehicles would have a detrimental impact on sustainability, because these vehicles spend time deadheading while in operation (*66*). Similar results are observed for out-of-vehicle travel time, echoing prior studies on the relationship between ride-sourcing and public transit (*67*). This could stem from the tendency for travel time and cost considerations (*68*) to influence the decision to use shared ride-sourcing.

**Table 5.** Direct and Cross Elasticity with Respect to Key Variables (Sample Averages)

Variable	Drive yourself	Driven by someone you know	Public transit	Exclusive ride-sourcing	Shared ride-sourcing	Taxi	Bicycle	Walking
Parking cost ($)	−0.550^d^	0.321	0.414	0.414	0.413	0.414	0.288	0.336
Out-of-vehicle travel time (min)	0.022	0.028	−0.186^d^	0.118	0.105	0.114	0.073	0.050

d Denotes direct elasticity values.

### Comparison across Income Groups

**Figure 7.**
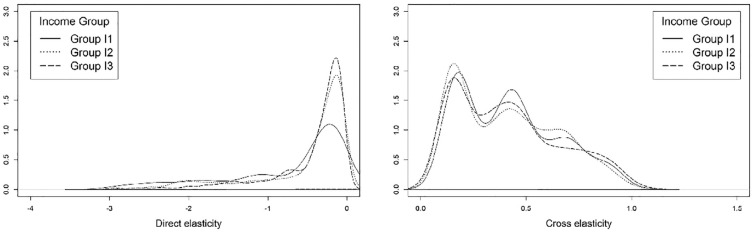
Elasticity of the *drive yourself* (left) and *public transit* (right) modes with respect to parking cost, by income group.

**Figure 8.**
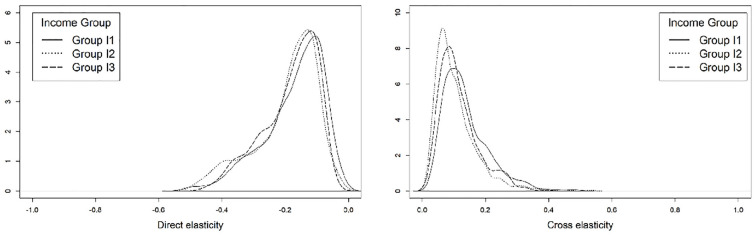
Elasticity of the *public transit* (left) and *exclusive ride-sourcing* (right) modes with respect to out-of-vehicle travel time, by income group.

To compare the direct and cross elasticities of the variables of interest, three income categories were defined: households earning less than $50,000 annually (group I1); households earning between $50,000 and $100,000 annually (group I2); and households earning over $100,000 annually (group I3). In the interests of brevity, only the elasticities of public transit and private vehicles/ride-sourcing are discussed. Kernel density plots are used to compare the distribution of elasticities across the groups. As shown in Figure 7, the lowest income group shows a greater sensitivity to an increase in parking costs than the other income groups. Additionally, individuals from income group I3 are slightly more likely to shift to public transit in response to an increase in the cost of parking. Such a shift could have a detrimental impact on lower-income households, because previous research has shown that the competitive housing market in the study area has resulted in these households moving to areas with lower levels of transit accessibility (*69*). As Figure 8 shows, an increase in out-of-vehicle travel time was found to have a slightly greater impact on the likelihood of using transit as household income level increases.

Interestingly, the cross elasticity for exclusive ride-sourcing in relation to transit out-of-vehicle travel time is greater for group I1 than for groups I2 and I3. This could be a reflection of the growth in the generation of ride-sourcing trips in lower-income areas that was observed pre-pandemic, or it could be the result of the complementary role that ride-sourcing can play in areas with lower transit accessibility (*70*). The results highlight the potential for transportation demand management strategies to have varied impacts across income groups.

### Comparison across Age Groups

**Figure 9.**
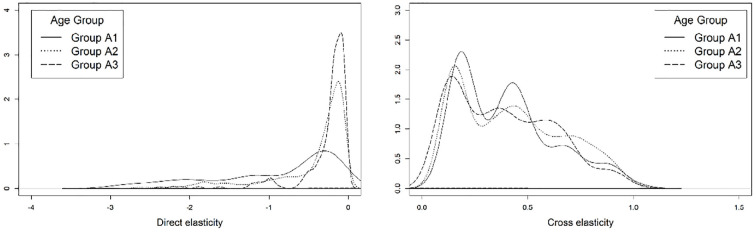
Elasticity of the *drive yourself* (left) and *public transit* (right) modes with respect to parking cost, by age group.

**Figure 10.**
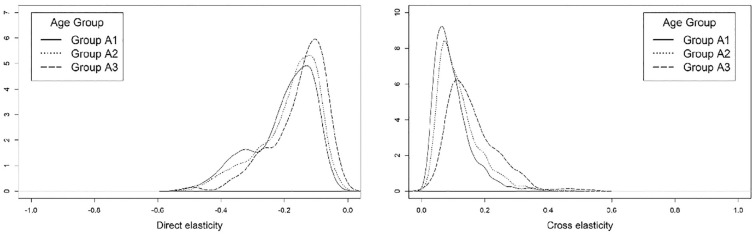
Elasticity of the *public transit* (left) and *exclusive ride-sourcing* (right) modes with respect to out-of-vehicle travel time, by age group.

Similar to the analysis of elasticities across different income groups, three age categories were defined: those aged 30 and younger (group A1); those between the ages of 31 and 64 (group A2); and those aged 65 and older (group A3). As shown in Figure 9, the sensitivity to parking costs when a person is driving appears to be lower for older age groups. This may stem from the tendency of transit and active mode usage to be more prevalent among younger individuals and the tendency for older individuals to favor travel by private vehicle (*71*). The likelihood of using public transit in response to an increase in parking costs was relatively consistent across the three age groups. However, the mean and median cross elasticity of group A3 were slightly lower than that of groups A1 and A2 (see Figure 9). This could stem from factors other than travel cost (e.g., access time and distances) influencing the decision to use public transit among seniors (*72*). Similarly, the sensitivity of an individual to the out-of-vehicle travel time when using public transit services also appears to be lower for older age groups (see Figure 10). Because older individuals tend to be less likely to use active modes, this could result from the relative lack of alternative travel modes that are available to seniors. However, on average, the A3 group also has the greatest likelihood of using exclusive ride-sourcing in response to an increase in out-of-vehicle travel time. Despite ride-sourcing use being more prevalent among younger individuals, there is evidence that ride-sourcing is also used by seniors (particularly those who are younger and have higher levels of educational attainment) (*73*). Although this result is somewhat counterintuitive, it could stem from the relatively low probability of older individuals choosing exclusive ride-sourcing (the average choice probabilities were 7.0%, 4.2%, and 2.2% for groups A1, A2, and A3, respectively).

## Discussion

The results shed light on the factors influencing mode choice decisions in the post-pandemic period, defined in this study as the period of time during which COVID-19 is no longer a public health threat. In particular, the results shed light on the influence (or lack thereof) of pandemic-related factors on anticipated post-pandemic mode choices for non-commuting trips. Similar to studies on mode choices during the pandemic, the findings suggest that perceptions of risk have the potential to influence the decision to used shared modes post-pandemic. Moreover, the frequency with which a mode was used pre-pandemic was also found to influence post-pandemic mode choices. However, the variables corresponding to health and safety measures were not found to have a statistically significant influence on post-pandemic mode choices. Overall, these results suggest that post-pandemic mode choices could resemble pre-pandemic mode choices, with discrepancies between the two being driven by lingering perceptions of risk and a reluctance to use shared modes.

In addition, the results offer insights into the extent to which pre-pandemic determinants of mode choices influence post-pandemic mode choice decisions. Overall, it appears that sociodemographic and level-of-service attributes influence post-pandemic mode choice decisions to a greater extent than perceptions of risk and other pandemic-related factors. However, the latter two still have the potential to influence the decision to use shared modes in the post-pandemic period. This information can help inform efforts to examine whether the changes in travel mode choices resulting from the pandemic, namely, an increase in private vehicle usage and a reduction in transit ridership, will persist post-pandemic. Moreover, these findings can help inform the development of policies that aim to address this trend and help encourage the use of more sustainable modes of travel.

Based on the outcomes of pre-pandemic studies and the results of this study, an increase in parking costs could help mitigate the increase in private vehicle use that occurred as a result of the pandemic. However, the implementation of such an increase must account for the modes that are available to trip-makers. In the absence of viable alternative modes of travel, individuals may opt to bear the increased costs or change their destination. Moreover, a reduction in driving in response to an increase in parking costs has the potential to increase ride-sourcing usage, which would not result in a reduction in vehicle kilometers traveled. This was exemplified in the results of this study, because the possession of a driver’s license increased the likelihood of an individual choosing exclusive ride-sourcing and the reluctance to use shared modes reduced the likelihood of an individual choosing transit. Such a shift could be mitigated through the introduction of a surcharge for trips made to areas with high transit accessibility, although this policy could have a disproportionate impact on lower-income individuals. Furthermore, the results of the elasticity analysis show that an increase in parking costs would have the greatest impact on individuals from lower-income households, which could have an adverse impact on their mobility and accessibility. Ensuring that other modes, particularly public transit, provide a reasonable level of mobility and accessibility is also an important consideration with regard to helping older members of the population retain sufficient levels of mobility and avoid transportation disadvantage (*4*). To help encourage the use of public transit, parking costs could be increased in areas with relatively high transit accessibility, with the additional parking revenues being used to help fund high-frequency services.

Additionally, the findings of this study suggest that transit usage could be less prevalent in the post-pandemic period than it was in the pre-pandemic period. This decline in transit usage could pose several challenges, particularly if trips that were previously made using transit are made by private vehicles, ride-sourcing, and taxis in the post-pandemic period. Although the implementation of mask mandates and the disinfection of vehicles were not found to influence the decision to use transit, the results suggest that reducing overcrowding could help mitigate a decline in transit use by assuaging concerns about the use of shared modes. This could be achieved by increasing the frequency of transit services, although this would likely require additional funding given the decline in fare revenues caused by the pandemic. Similarly, transit agencies could make use of data obtained through automated fare collection and automated passenger counting systems to make real-time information on crowding levels on individual transit vehicles publicly available. Both measures can help rebuild public confidence in public transit, which is a key factor in helping transit ridership recover to pre-pandemic levels (*74*).

Previous studies have found evidence that newer generations of seniors (i.e., those over 65) are more likely to rely on private vehicles for mobility than their predecessors (*75*). As these individuals continue to age, they will eventually be unable to drive. This would have a negative impact on their accessibility, and by extension, their well-being, particularly if they live in areas with poor walkability and transit accessibility. Furthermore, the results of this study suggest that in the post-pandemic period, older individuals are more likely to drive and less likely to use taxis and ride-sourcing. The reduction in mobility and accessibility that results from the loss of the ability to drive can be mitigated by improvements in both the coverage and frequency of transit services. As indicated by the model results, longer waiting and walking times discourage the use of public transit. Consequently, the viability of public transit could potentially be improved by increasing the coverage of the transit network (to reduce access and egress times), coordinating transfers between routes, and increasing services on key routes (to reduce waiting time). However, given the loss of transit ridership that resulted from the pandemic, and the relationship between fare revenue and operating costs, implementing these strategies would probably require additional funding from local governments. Additionally, the introduction of parking restrictions along high-frequency transit corridors, the implementation of dedicated transit lanes, and the use of transit signal priority can help improve the reliability of transit services. These improvements could assist in encouraging public transit use, which would help reduce waiting times and improve the perception of transit services.

## Conclusions and Future Work

This study presented the results of an investigation into the determinants of anticipated post-pandemic mode choices for non-commuting trips among residents of the GTA. Using data from a web-based survey, the study developed a random parameter mixed logit model based on information collected through a series of SP experiments. The model was used to compute the direct and cross elasticity of key variables, which were then compared across income and age groups. The model results highlight the influence of sociodemographic and level-of-service attributes on post-pandemic mode choices. The results also suggest that pre-pandemic travel habits offer insights into post-pandemic modal preferences. Specifically, the frequent pre-pandemic use of a mode increased the likelihood that it was chosen in the SP experiments. In addition, the results suggest that certain individuals may still be reluctant to use shared modes in the post-pandemic period. Furthermore, the results of the elasticity analysis show that the effects of potential transportation demand management strategies would differ across income and age groups. Although the study offers insights into approaches that policymakers could take to combat the increase in private vehicle use and reduction in transit use that have resulted from the pandemic, it also highlights the need to ensure that a sufficient level of mobility and accessibility can be maintained without the use of a private vehicle.

The key limitation is that the results are based on an analysis of SP data pertaining to post-pandemic mode choices. In addition to the long-standing issues associated with the hypothetical nature of SP data (*62*), the survey defined the post-pandemic period as the time when COVID-19 is no longer a public health threat. At the time of writing, it is still unclear whether COVID-19 will be eradicated or if it will become an endemic disease. If the latter were to happen, it would mean that the nature of the post-pandemic period would differ from the description provided to the respondents, which could affect the generalizability of the results. Additionally, the use of a web-based survey to collect the information could result in the participants having more familiarity with technology on average than the study area population, which has been found to affect the propensity to use ride-sourcing (*76*). These factors, coupled with the underrepresentation of certain sociodemographic attributes in the sample, could have an adverse impact on the extent to which the results of this study can be generalized to the population of the study area.

Future studies could build on the work presented in this study by incorporating latent attitudinal variables into their analysis through the use of factor analysis, structural equation models, or the integrated choice and latent variable model. Doing so would allow the researcher to examine the impact of latent attitudinal factors and perceptions of risk on post-pandemic mode choices. Future studies could also estimate a similar model using the error component mixed logit formulation, which would allow more complex substitution patterns to be captured in the model. In addition, future studies could utilize SP experiments whose attribute values are pivoted off the attributes of trips reported by the respondents. Taking a pivoted approach to designing the experiments has been shown to assist in improving the behavioral realism of the responses, which can help address the issues stemming from the hypothetical nature of the data (*77*). Finally, future studies could extend the elasticity analysis presented in this paper to consider the intersection of factors such as race, gender, income, and residential location to gain deeper insights into the potential impact of transportation demand management strategies on disadvantaged groups.
